# Evaluating Effectiveness of the FiTeens Intervention for Health Behavior Change in Students: A Study Protocol

**DOI:** 10.3390/mps9040101

**Published:** 2026-07-01

**Authors:** Taavi Rand, Henri Tilga, Ángel Abós, Luis García-González, Sergio Diloy Peña, Rafael Burgueño-Mengibar, Andre Koka

**Affiliations:** 1 Institute of Sport Science and Physiotherapy, Faculty of Medicine, University of Tartu, Ujula 4, 51008 Tartu, Estonia; henri.tilga@ut.ee (H.T.); andre.koka@ut.ee (A.K.); 2 Faculty of Health and Sport Sciences, University of Zaragoza, 22002 Huesca, Spain; aabosc@unizar.es (Á.A.); lgarciag@unizar.es (L.G.-G.); 3 Faculty of Health Sciences, Public University of Navarre, 31006 Pamplona, Spain; sergio.diloy@unavarra.es; 4 Department of Didactics in Language, Art and Sport, University of Malaga, 29071 Malaga, Spain; rburgueno@uma.es

**Keywords:** physical activity, sleeping habits, screen time, intervention program, students, health behavior, FiTeens

## Abstract

(1) Background: Previous school-based interventions have addressed adolescent health behaviors such as physical activity, screen time, and sleep, but have predominantly targeted these behaviors independently rather than simultaneously. The Erasmus+ project FiTeens developed an integrated intervention combining theoretical content, videos, infographics, and interactive tasks to promote multiple health behaviors concurrently. The objective of the current article is to present the protocol for a school-based intervention study designed to examine the effects of the FiTeens program on adolescents’ physical activity, screen time, and sleep behaviors. We hypothesize that students receiving the FiTeens intervention will demonstrate increased physical activity, reduced screen time, and improved sleep outcomes compared with students in the control group. (2) Methods: Teachers will be introduced to the FiTeens tools prior to delivering the intervention to students in grades 5–9. Students will participate in an eight-week intervention program combining structured lessons and behavior-change challenges. Primary outcomes include changes in physical activity, screen time, and sleep duration and quality. Secondary outcomes include psychological determinants such as motivation and behavioral intentions. Data will be collected at baseline and at 1-, 3-, and 6-month follow-ups and analyzed using repeated measures ANOVA. (3) Expected results: The study will evaluate whether the intervention may contribute to improvements in health-related behaviors among adolescents, including increased physical activity, reduced screen time, and improved sleep outcomes. (4) Conclusions: The intervention based on FiTeens tools could have the potential to promote healthier lifestyle behaviors among students by increasing physical activity during leisure time, supporting the effective limitation of screen time and enhancing bedtime routines to improve sleep quality.

## 1. Introduction

Physical inactivity, excessive screen time, and insufficient sleep are common health concerns among adolescents and are associated with adverse physical and psychological outcomes [1,2,3,4,5,6,7,8]. These health behaviors are closely interrelated and represent important targets for school-based health promotion initiatives.

Problems associated with low physical activity, excessive screen time, and insufficient sleep can be addressed through various intervention programs [9]. A meta-analysis Indicated that the most effective interventions were school-based and multifaceted, incorporating different physical activities. Shorter interventions lasting 12–26 weeks appeared somewhat more effective than longer interventions (>26 weeks) [10]. Previous research has also highlighted the importance of involving teachers and parents in intervention delivery, consistent with the Creating Active Schools framework, which emphasizes the promotion of healthy lifestyles through the entire school community [11]. Although some interventions have focused on screen time reduction, many have primarily targeted dietary behaviors, with only limited emphasis on physical activity and other related health behaviors [12].

The current study is based on the Erasmus+ project “FiTeens: Promoting Physical Activity and Healthy Lifestyles among Sedentary Youth”, which developed a set of digital educational resources, including an e-book, a MOOC platform, and web-based health behavior challenges designed to promote healthy lifestyle behaviors among adolescents [13]. Unlike many previous interventions, the FiTeens program simultaneously targets physical activity, screen time, and sleep through a structured school-based approach combining teacher-led lessons, digital learning materials, and behavioral challenges. Furthermore, the intervention is grounded in Self-Determination Theory and the Theory of Planned Behavior, aiming to support motivation, behavioral intentions, and sustainable behavior change across multiple health behaviors simultaneously. By integrating digital resources directly into the educational process, the FiTeens intervention may provide a more comprehensive and ecologically valid approach for promoting healthy lifestyles among adolescents within real-world school settings.

The aim of the present protocol article is to describe the design, intervention procedures, outcome measures, and planned evaluation of a school-based FiTeens intervention designed to promote physical activity, reduce screen time, and improve sleep among adolescents.

## 2. Materials and Methods

### 2.1. Study Design

The study is designed as a quasi-experimental study with school-level allocation, involving one intervention school and one control school. Due to practical constraints in the school setting, randomization at the individual or school level was not feasible. This approach was chosen to minimize contamination between participants within the same school. As a result, potential school-level differences may influence the observed outcomes. To address this limitation, baseline characteristics will be compared between groups and considered when interpreting the findings. Nevertheless, residual confounding due to contextual differences between schools cannot be entirely ruled out. Therefore, caution should be exercised when generalizing the findings beyond the participating schools.

### 2.2. Participants

Participants of this study will be students from grades 5 to 9 (ages 11–15) recruited from general education schools in Estonia, with one school designated as the intervention group and one school designated as the control group.

Sample size estimation was conducted using G*Power 3.1 based on a repeated measures ANOVA design with two groups and four measurement occasions. Assuming a medium effect size (f = 0.25), an alpha level of 0.05, and a statistical power of 0.80, a minimum sample size of 128 participants was required (64 participants per group). To account for potential participant attrition during follow-up assessments, the target sample size was increased by approximately 40–50%. Dropout rates of 20–40% have been reported in previous school-based interventions involving adolescents, which informed the decision to increase the sample size [14].

Participants in the control group will continue with their regular school curriculum and will not receive the FiTeens intervention during the study period.

### 2.3. Ethical Considerations, Consent, and Permissions

The study will be conducted in accordance with the Declaration of Helsinki and the principles of Good Clinical Practice. Ethical approval was obtained from the Research Ethics Committee of the University of Tartu (code: 1/M-6, 27 January 2026). Participation in the study is voluntary, and participants may withdraw at any time without consequences. Written informed consent will be obtained from both students and their legal representatives prior to participation. As a substantial proportion of the target population consists of non-native Estonian speakers, all study materials and consent forms will be provided in both Estonian and Russian.

### 2.4. Intervention

**Figure 1:**
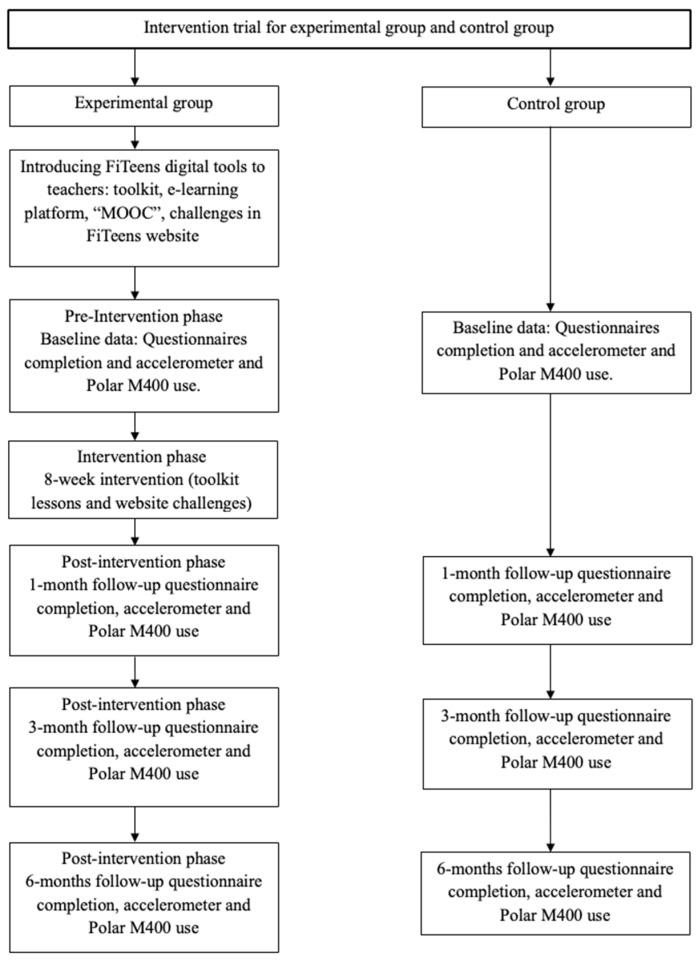
Main trial intervention design.

Prior to delivering the intervention to 5–9th grade students, teachers will first be introduced to FiTeens digital tools, including the toolkit, MOOC e-learning platform and challenges available on the FiTeens website, during 5 h intensive course. After collecting the baseline data, students will undergo an eight-week intervention program comprising lessons delivered by teachers on health-related topics combining behavior challenges provided through the FiTeens toolkit (Figure 1).

**Figure 2:**
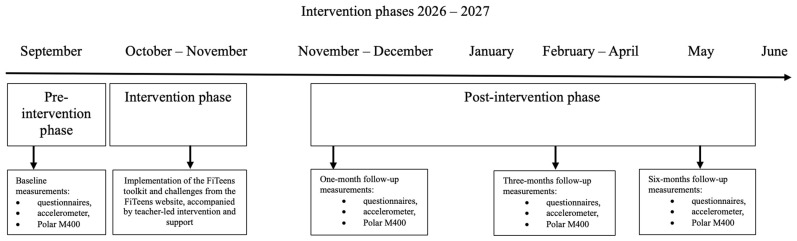
Intervention phases in stage 2.

During the intervention students will undergo an eight-week program including lessons based on the content from toolkit and challenges in FiTeens website: www.fiteens.eu. Each session is based on a predefined thematic focus (physical activity, screen time, or sleep) and includes a combination of theoretical input, group discussion, and practical activities designed to promote behavior change. The study consists of pre-intervention phase, intervention phase and post-intervention phase (Figure 2).

#### 2.4.1. Pre-Intervention Phase

Prior to the implementation of the intervention program, baseline data will be collected through questionnaire administration, the use of accelerometers and Polar M400 smartwatch worn for seven consecutive days. The Polar M400 smartwatch is used as an objective measure of sleep duration. The devices are randomly distributed among students and worn on the wrist for a period of seven days. In addition, students will fill out sleep diary. Accelerometers will also be distributed, which each participant will wear on the hip for a period of seven days.

#### 2.4.2. Intervention Phase

During the intervention phase, students will participate in an 8-week school-based program consisting of weekly teacher-led lessons supported by the FiTeens educational resources. Each lesson will last 90 min and follow a standardized structure including an introduction, main activity, and conclusion. Students will receive a learning portfolio focusing on physical activity, screen time, and sleep, and will complete weekly home-based challenges available through the FiTeens platform. Challenge completion and reflections will be documented using a challenge diary and discussed during subsequent lessons. The intervention incorporates behavior change techniques such as goal setting, self-monitoring, guided reflection, and group discussion. An overview of the intervention content is presented in Figure 2.

#### 2.4.3. Intervention Fidelity

To ensure consistency in the delivery of the intervention, teachers will be provided with standardized materials, including the FiTeens toolkit, structured lesson plans, student learning portfolio, intervention manual, and standardized PowerPoint presentations prepared for each lesson. Teachers will receive an initial introduction to the FiTeens digital tools and intervention procedures prior to implementation. Each lesson follows a predefined structure consisting of an introduction, main activity, and conclusion, which supports consistency across sessions and schools. Adherence to the intervention will be monitored through student-completed challenge diaries and regular reflection activities conducted at the beginning of each session, providing insight into student participation in assigned challenges. Although formal observation of teaching sessions is not included, the use of standardized materials, unified PowerPoint presentation formats for each lesson, and structured implementation procedures is intended to support intervention fidelity.

#### 2.4.4. Post-Intervention Phase

Post-intervention data collection is conducted at 1, 3 and 6 months after the intervention and included questionnaire completion and the use of an accelerometer and Polar M400 for seven consecutive days.

### 2.5. Psychological and Behavioral Measures

Students will complete questionnaires assessing motivation and behavioral determinants related to physical activity, screen time, sleep habits, and sedentary behaviors at baseline and at one-, three-, and six-month follow-up assessments. Prior to the main intervention study, the construct validity of translated and adapted questionnaires will be examined using data collected from an independent pilot sample of adolescents. Internal consistency of all multi-item scales will be assessed using Cronbach’s alpha coefficients. The primary outcomes will be changes in health-related behaviors, including physical activity, screen time, and sleep duration and quality, which represent the main targets of the FiTeens intervention. These outcomes will be assessed using both objective measures (accelerometers and Polar M400 smartwatch-based sleep assessment) and subjective questionnaire-based measures. Secondary outcomes will include psychological determinants of behavior based on Self-Determination Theory and the Theory of Planned Behavior, including motivation, behavioral intentions, attitudes, subjective norms, and perceived behavioral control related to physical activity, screen time, and sleep behaviors.

#### 2.5.1. Motivation Assessment

Motivation will be assessed using the Motivation to Limit Screen-time Questionnaire (MLSQ) [15] and a validated physical activity motivation questionnaire based on Self-Determination Theory [16]. The MLSQ will be used to assess motivation related to screen time and, in an adapted form, sleep-related behaviors. The physical activity motivation questionnaire assesses autonomous motivation (intrinsic motivation and identified regulation) and controlled motivation (introjected and external regulation) toward leisure-time physical activity. Together, these instruments assess autonomous motivation, controlled motivation, and amotivation across the three target health behaviors. The adapted sleep-related version of the MLSQ will retain the original theoretical structure of the instrument. Construct validity and internal consistency of the adapted measures will be evaluated prior to the main study. Detailed information regarding questionnaire items and response for mats is presented in Appendix A (Table A1).

#### 2.5.2. Behavioral Determinants Assessment

Behavioral determinants will be assessed using a questionnaire based on the Theory of Planned Behavior. The questionnaire measures attitudes, subjective norms, perceived behavioral control, and intentions related to physical activity, screen time, and sleep. Responses are recorded using seven-point Likert-type scales. Higher scores indicate stronger endorsement of the respective construct [17,18]. Detailed information regarding the questionnaire items and response formats is presented in Appendix A (Table A1).

#### 2.5.3. Sleep Hygiene Questionnaire

The questionnaire is aimed at children/adolescents between 6 and 17 years of age. The questionnaire evaluates sleep-related behaviors and habits during the previous two weeks, including bedtime routines, caffeine consumption, screen use before bedtime, and other behaviors known to influence sleep quality and duration. Participants respond to items reflecting the frequency of specific behaviors, providing an overall indication of sleep hygiene practices [19]. Detailed information regarding the questionnaire items and response formats is presented in Appendix A (Table A1).

#### 2.5.4. Youth Leisure-Time Sedentary Behavior Questionnaire

The participants are asked to think back over the previous week and report the estimated average time devoted to each behavior during weekdays and weekend days, separately. The average time per day spent on each behavior and composite category was calculated as follows: [(weekday_time × 5) + (weekend_time × 2)]/7 [20]. Detailed information regarding the questionnaire items and response formats is presented in Appendix A (Table A1).

#### 2.5.5. Leisure Time Exercise Questionnaire

Subjective leisure-time physical activity is assessed using the Leisure Time Exercise Questionnaire [21]. The instrument is designed to evaluate participation in vigorous physical activity during leisure time and provides an indicator of habitual physical activity behavior outside the school setting. Detailed information regarding the questionnaire items and response formats is presented in Appendix A (Table A1).

#### 2.5.6. ActiGraph GT3X Accelerometer

Objective physical activity is assessed using ActiGraph GT3X accelerometers (ActiGraph LLC, Pensacola, FL, USA). The accelerometers are used to measure moderate-to-vigorous intensity leisure-time physical activity in adolescents. Accelerometer data will be processed using established criteria. A valid day will be defined as at least 10 h of wear time. Non-wear time will be identified as periods of at least 60 consecutive minutes of zero counts, allowing for short interruptions. Participants with at least 4 valid days, including at least one weekend day, will be included in the analysis. Missing data will be handled according to standard procedures, and only valid wear-time data will be included in the final analyses. Accelerometer data will be collected using a sampling epoch length of 15 s. Physical activity intensity will be classified using established cut-off points for adolescents (e.g., moderate-to-vigorous physical activity thresholds based on counts per minute). Data will be processed using appropriate software (e.g., ActiLife software, version 6.13.3, ActiGraph LLC). These criteria are consistent with commonly used protocols in adolescent physical activity research. Participants are instructed to wear the accelerometers on the hip for seven consecutive days in baseline, 1-month follow-up, 3-month follow-up and in 6-month follow-up.

#### 2.5.7. Polar M400 Smartwatch

Objective sleeping duration is assessed using Polar M400 smartwatch (Polar Electro Oy, Kempele, Finland). The Polar M400 smartwatch is used to measure sleep duration in adolescents. Sleep duration will be assessed using the Polar M400 wrist-worn device. The device estimates sleep based on accelerometer-derived movement data, identifying periods of inactivity that correspond to sleep. Sleep duration is calculated automatically by the device as the time between sleep onset and wake-up, determined through changes in movement patterns and wrist activity levels. The extracted variable used in the analysis will be total nightly sleep duration (in hours), as recorded by the device. The Polar M400 smartwatch provides an estimate of sleep duration based on accelerometer-derived movement data and has been used as a practical tool for assessing sleep duration in free-living conditions. It should be noted that the device primarily estimates sleep duration rather than detailed sleep architecture or sleep quality. The Polar M400 smartwatch estimates sleep based on movement patterns and does not provide detailed information on sleep stages or sleep quality comparable to gold-standard methods such as polysomnography. Therefore, the objective sleep-related outcome in this study is limited to sleep duration. Participants are instructed to wear the device on the wrist for seven consecutive days at baseline, 1-month follow-up, 3-month follow-up, and 6-month follow-up.

### 2.6. Statistical Analysis

All statistical analyses will be conducted using IBM SPSS Statistics for Windows, Version 29.0 (IBM Corp., Armonk, NY, USA) and JASP, Version 0.17.1 (JASP Team, Amsterdam, The Netherlands). Descriptive statistics will be calculated for all study variables. The primary statistical analysis will be performed using repeated measures ANOVA, which was selected because it is appropriate for evaluating within-subject changes across repeated measurement occasions and between-group differences over time in the present study. The analysis will examine the main effects of group and time, as well as the group × time interaction effect for physical activity, screen time, and sleep-related outcomes across four measurement points (baseline, 1-, 3-, and 6-month follow-up assessments). Baseline measurements will be included in the repeated measures design to evaluate changes over time within and between groups. Independent samples *t*-tests will additionally be used to examine baseline differences between the intervention and control group, as well as differences between study completers and dropouts at baseline. As schools are allocated to intervention and control conditions at the school level, baseline differences between groups will be examined prior to the main analyses and considered when interpreting the findings. Potential school-level effects and residual confounding cannot be completely excluded and should therefore be considered when interpreting the study findings. Prior to the main analyses, data will be screened for missing values, and the extent and pattern of missingness will be examined and reported. Missing data will be addressed using multiple imputation procedures prior to analysis. An intention-to-treat approach will be applied, including all participants as originally allocated to their groups. Dropout rates and reasons for attrition will be reported and examined. Potential confounding variables, such as age and gender, will be considered where appropriate.

## 3. Expected Results

The present protocol article describes the design and planned evaluation of a school- based intervention based on the FiTeens project, aimed at promoting physical activity, reducing screen time, and improving sleep among adolescents. It is anticipated that participation in the intervention may be associated with favorable changes in physical activity levels, sleep duration, and screen time compared with the control group at the one-month follow-up. Previous intervention studies have demonstrated that school-based and digitally supported programs can contribute to improvements in adolescents’ physical activity levels and related health behaviors [22,23]. Similarly, prior research has shown that interventions targeting sedentary behavior may reduce screen time, particularly when behavioral and motivational components are included [24]. In addition, sleep-focused interventions have been associated with improvements in sleep duration and sleep hygiene among adolescents [25]. Furthermore, previous research suggests that the maintenance of behavior change over time is influenced by motivational and environmental factors [26], indicating the potential for favorable outcomes to be maintained at the 3- and 6-month follow-ups. These potential changes will be assessed using both objective and subjective measures. In addition, the study will explore whether participation in the intervention is associated with changes in psychological constructs such as motivation, behavioral intentions, attitudes, and perceived behavioral control. However, the study is not designed to test complex mediation models, and, therefore, interpretations regarding underlying mechanisms will be made with caution. If the intervention demonstrates favorable outcomes, it may provide a basis for further research on integrated, school-based interventions targeting multiple health behaviors. However, conclusions regarding long-term effectiveness, scalability, and cost-effectiveness will require additional empirical evidence. The study may also provide insight into the feasibility and acceptability of implementing digitally supported health behavior interventions within everyday school settings.

## Data Availability

Data from the experimental study will be available in the Open Science Framework (OSF) data repository.

## Appendix A

**Table A1:** Overview of the questionnaires and measurement instruments.

Construct	Instrument (Source)	Subdimension	Example Item	Response Format
Motivation to Limit Screen-time Questionnaire (MLSQ)	Lubans et al. 2013 [15]	Autonomous motivation	“I try to [target behavior] because I believe it is important for my health”	1–7 Likert (1 = untrue, 7 = true)
		Controlled motivation	“I try to [target behavior] because I would feel guilty if I did not”	1–7 Likert (1 = untrue, 7 = true)
		Amotivation	“I do not see any reason to [target behavior]	1–7 Likert (1 = untrue, 7 = true)
Physical Activity Motivation Questionnaire	Kalajas-Tilga et al. 2021 [16]	Autonomous motivation	“I am physically active during my leisure time because it is fun”	1–7 Likert (1 = untrue, 7 = true)
		Controlled motivation	“I am physically active during my leisure time because I would feel bad about myself if I were not physically active”	1–7 Likert (1 = untrue, 7 = true)
		Amotivation	“I do not see any reason to be physically active during my leisure time”	1–7 Likert (1 = untrue, 7 = true)
Student’s behavioral determinants (TPB)	Ajzen. 1991 [18]	Behavioral intention	“I intend to engage in [target behavior] during next 4 weeks”	1–7 Likert (1 = untrue, 7 = true)
		Attitudes	“Engaging in [target behavior] during next week is…”	1–7 Likert (1 = useless, 7 = useful; 1 = bad, 7 = good; 1 = unpleasant, 7 = pleasant)
		Subjective norms	“Most people who are important to me think I should engage in [target behavior] during next 4 weeks.”	1–7 Likert (1 = disagree, 7 = agree)
		Perceived behavioral control	“How much control do you have over engaging in [target behavior] during next 4 weeks?”	1–7 Likert (1 = very little control, 7 = complete control)
Sleep Hygiene Questionnaire	Paulsrud et al. 2023 [19]	Sleep behavior habits and behaviors	“During the last hour before going to bed, how often do you … drink caffeine drinks”	Never–more than 5x a week
Youth Leisure-time Sedentary Behavior Questionnaire	Cabanes-Sanchez et al. 2018 [20]	Screen time habits and behaviors	“During previous week how much time did you spent watching TV/videos/DVDs?”	Weekday 0 min–5 h or more; Weekend day 0 min–5 h or more
Leisure Time Exercise Questionnaire	Godin et al. 1985 [21]	Leisure time exercise	“How often have you engaged in vigorous physical activity during your leisure time over the past four weeks for at least 20 min at a time?”	1–6 Likert (1 = never, 6 = most days of the week)

Note. The example items presented in this table are intended to illustrate the assessed constructs and do not represent all questionnaire items. For the MLSQ the term [target behavior] refers to screen time or sleep and for TPB measures, the term [target behavior] refers to physical activity, screen time, or sleep, depending on the behavioral domain being evaluated.
